# Antibody feedback regulation

**DOI:** 10.1111/imr.13377

**Published:** 2024-08-23

**Authors:** Birgitta Heyman

**Affiliations:** ^1^ Department of Medical Biochemistry and Microbiology Uppsala University, (BMC) Uppsala Sweden

**Keywords:** CD23, epitope masking, Fc gamma receptors, IgE, immune complexes

## Abstract

Antibodies are able to up‐ or downregulate antibody responses to the antigen they bind. Two major mechanisms can be distinguished. Suppression is most likely caused by epitope masking and can be induced by all isotypes tested (IgG1, IgG2a, IgG2b, IgG3, IgM, and IgE). Enhancement is often caused by the redistribution of antigen in a favorable way, either for presentation to B cells via follicular dendritic cells (IgM and IgG3) or to CD4^+^ T cells via dendritic cells (IgE, IgG1, IgG2a, and IgG2b). IgM and IgG3 complexes activate complement and are transported from the marginal zone to follicles by marginal zone B cells expressing complement receptors. IgE–antigen complexes are captured by CD23^+^ B cells in the blood and transported to follicles, delivered to CD8α^+^ conventional dendritic cells, and presented to CD4^+^ T cells. Enhancement of antibody responses by IgG1, IgG2a, and IgG2b in complex with proteins requires activating FcγRs. These immune complexes are captured by dendritic cells and presented to CD4^+^ T cells, subsequently helping cognate B cells. Endogenous feedback regulation influences the response to booster doses of vaccines and passive administration of anti‐RhD antibodies is used to prevent alloimmunization of RhD‐negative women carrying RhD‐positive fetuses.

## 1 INTRODUCTION

Antibodies, forming an immune complex with their specific antigen, can up‐ or downregulate the antibody response to this antigen. The phenomenon is called antibody feedback regulation and has been known for more than 100 years.^1^, ^2^ Feedback regulation is potent and can cause over 99% suppression or a several 100‐fold enhancement of the antibody response. Regulation is limited to the antigen to which the antibody binds, but may affect other epitopes than those directly targeted by the regulating antibody.

The ability of IgG antibodies to suppress responses to erythrocytes has been applied successfully in humans to prevent hemolytic disease of the fetus and newborn (HDFN).^3^ RhD‐negative women carrying RhD‐positive fetuses may produce IgG anti‐RhD when transplacental bleedings have occurred. These antibodies are actively transported over the placenta and can destroy fetal erythrocytes. Starting in the 1960s,^4^ anti‐RhD antibodies from immunized serum donors are administered to mothers at risk, either during pregnancy or immediately after delivery. This treatment prevents further alloimmunization of the women and has significantly decreased the incidence of HDFN.^3^ Other examples of antibody feedback regulation in humans is the inhibition of responses to booster doses of vaccines by preexisting antibodies.^5^, ^6^, ^7^ Similarly, virus‐specific IgG from vaccinated or naturally infected mothers, transferred over the placenta, can be a problem in vaccinations of infants during the first months after birth.^8^, ^9^

The mechanisms behind antibody feedback regulation have been the subject of research for decades. The most common approach has been to passively administer specific antibodies together with antigen in physiological salt solutions to animals, usually mice, and analyze the difference in immune responses between such animals and controls receiving antigen alone. Studies can roughly be divided into three phases. Initially, the regulatory effects of passively transferred whole immune serum were studied in rabbits, guinea pigs, rats, and mice (reviewed in^10^). A second phase (reviewed in^11^, ^12^, ^13^, ^14^) began when serum could be separated into different isotypes and when monoclonal antibody technology facilitated production of large quantities of well‐defined antibodies with known isotypes and specificities. Mutated monoclonal antibodies with a defined loss of effector functions, or able to block Fc and complement receptors in vivo, were also useful. However, gene‐targeted KO (knock‐out) mice soon became a better tool in elucidating the various feedback pathways. Currently, a third phase can be discerned (reviewed in^15^). Advanced methods such as fate mapping, Ig knock‐in mice, and cloning and sequencing of antibodies produced by defined B cell subtypes, have allowed in‐depth studies of how feedback regulation affects germinal center and memory B cells in mice and humans.^5^, ^6^, ^7^, ^16^, ^17^, ^18^, ^19^

Several explanations for how antibodies exert their dramatic regulatory effects on antibody responses have been proposed. Hypotheses to explain enhancement of antibody responses are (i) increased transport and concentration of antigen to areas in spleen or lymph nodes where interaction between antigen, B cells, and T cells takes place; (ii) increased uptake of immune complexes by antigen‐presenting cells, leading to increased activation and proliferation of T helper cells and increased B cell help; or (iii) augmented B‐cell signaling following co‐crosslinking of BCR and the complement receptor 2 (CR2)/CD19/CD81 co‐receptor complex. Suppression may hypothetically be induced by (i) antibodies masking epitopes on the antigen, thus preventing B‐cell recognition, (ii) efficient elimination/clearance of the antigen, (iii) negative B‐cell signaling following co‐crosslinking of the inhibitory FcγRIIB and BCR, (iv) complement‐mediated lysis of antigen, or (v) trogocytosis (antigen modulation or antigen loss) caused by antibodies binding to a certain epitope and physically removing it from the antigen.

The primary focus in my laboratory has been to elucidate the mechanisms behind the dramatic feedback regulatory effects of different antibody classes and subclasses in vivo and this will also be the focus of this review. Unless otherwise stated, only in vivo studies will be discussed. For additional references, please see previous reviews.^11^, ^12^, ^13^, ^14^ An overview of the key findings about antibody feedback regulation is shown in Table 1.

**TABLE 1:**
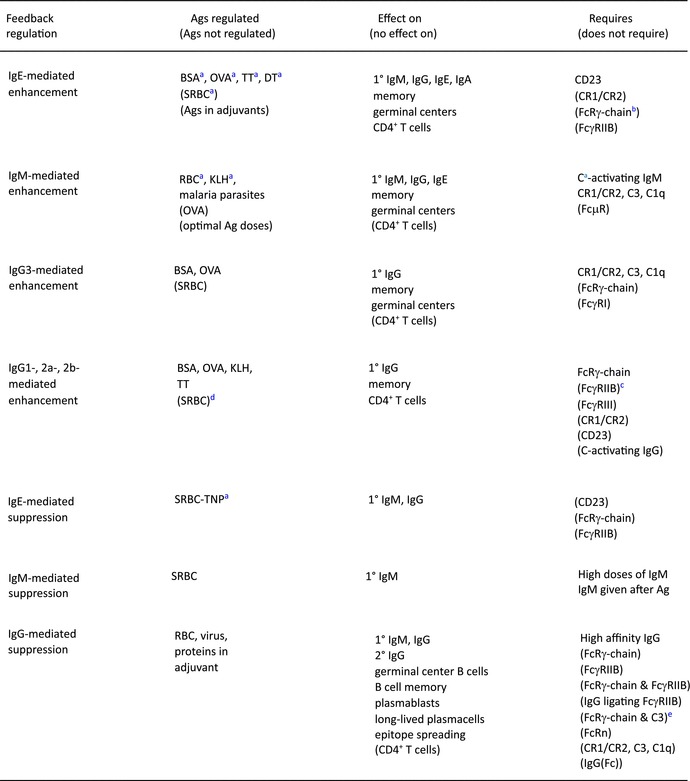
Key findings in antibody feedback regulation.

^a^ Bovine serum albumin (BSA), ovalbumin (OVA), tetanus toxoid (TT), diphtheria toxin (DT), sheep red blood cells (SRBC), red blood cells (RBC), keyhole limpet hemocyanin (KLH), trinitrophenyl (TNP), complement (C).

^b^ FcRγ‐chain knock‐out mice lack FcεRI, FcγRIII, and FcγRIV.

^c^ In the absence of FcγRIIB, these IgG subclasses induce an enhanced enhancement.^43^, ^44^

^d^ IgG usually suppresses SRBC responses but enhancement has been observed.^57^, ^105^, ^106^, ^107^

^e^ Suppression of responses to SRBC,^142^ but not to allogeneic mouse erythrocytes,^141^ works well in double knock‐out mice lacking both FcRγ‐chain and C3.

## 2 ANTIBODY FEEDBACK ENHANCEMENT

IgM, IgG1, IgG2a, IgG2b, IgG3, IgE, and IgA are all able to enhance antibody responses.

### 2.1 Enhancement by IgA

Few studies on IgA‐mediated enhancement have been published. Monoclonal IgA anti‐DNP (2,4‐dinitrophenyl) administered in complex with KLH (keyhole limpet hemocyanin)‐DNP can induce memory B cells,^20^ whereas the same monoclonal IgA had no effect on primary IgG anti‐KLH responses.^21^ IgA does not activate complement and is mainly exerting its effects at mucosal sites and may therefore not have a major influence on immune responses to antigens administered via conventional routes.

### 2.2 Enhancement by IgE

The first description of IgE‐mediated enhancement of antibody responses in vivo was published in 1993.^22^ What prompted us to investigate the possibility of IgE as an immunoregulatory isotype was a publication from the Bruijnzeel‐Koomen laboratory suggesting that IgE was specialized in antigen capture.^23^ In vitro, B cells binding IgE–antigen via CD23 (low‐affinity Fc receptor for IgE) had been shown to internalize and present antigenic peptides to CD4^+^ T cells,^24^ a finding later confirmed by others.^25^, ^26^, ^27^, ^28^, ^29^ Using a panel of monoclonal TNP‐specific IgE antibodies, we found that IgE administered to mice with BSA (bovine serum albumin)‐TNP (2,4,6‐trinitrophenyl) caused an up to 132‐fold stronger IgG anti‐BSA response than seen in mice given BSA‐TNP alone.^22^ The entire effect was abolished by blocking CD23 with a monoclonal antibody.^22^

IgE‐mediated enhancement is antigen‐ but not epitope‐specific.^22^, ^30^, ^31^ Monoclonal TNP‐specific IgE enhances carrier responses after administration together with BSA‐TNP,^22^, ^30^, ^31^, ^32^, ^33^, ^34^ OVA (ovalbumin)‐TNP,^29^, ^30^, ^31^, ^35^, ^36^ tetanus toxin‐TNP,^30^, ^31^ and diphtheria toxin‐TNP.^31^ Monoclonal IgE anti‐DNP^37^ as well as monoclonal IgE anti‐OVA^38^ also enhance antibody responses. In contrast, IgE anti‐TNP did not enhance responses to SRBC‐TNP,^30^ implying that only the response to small proteins is affected.

The lowest dose shown to induce enhancement is 10 μg IgE per mouse.^22^, ^30^ Fifty μg IgE was able to enhance responses to as little as 2 μg BSA‐TNP.^31^ IgE administered intravenously enhances IgG responses to antigen given intravenously or intraperitoneally during several weeks.^32^ Enhancement against subcutaneously administered antigen can only be seen during the first week and IgE was unable to enhance responses to antigens in complete Freund's adjuvant.^32^ IgE must be administered within 1 h of the antigen for optimal enhancement.^30^ Primary IgG responses are increased already 3 days after immunization,^32^ peaks after 2 weeks and are sustained during at least 5 weeks.^22^, ^30^, ^32^

IgE enhances primary IgG responses, including IgG1^29^, ^30^, ^37^, ^39^ and IgG2a.^29^, ^30^, ^39^ Primary IgE,^30^, ^32^, ^39^ IgA,^32^ and IgM^32^ responses are also enhanced during the first week. IgE is a potent inducer of germinal centers^36^ and immunological memory.^30^ Mice primed with IgE and antigen and boosted with antigen alone after 3 months had more than a 100‐fold higher IgG response than mice primed with antigen alone.^30^ IgE enhances activation and proliferation of T helper cells as shown in mice adoptively transferred with DO11.10 transgenic OVA‐specific CD4^+^ T cells and immunized with IgE anti‐TNP and OVA‐TNP.^29^, ^35^, ^40^ T‐cell proliferation was dependent on the presence of CD23^+^ B cells.^35^

IgE enhances antibody responses in all normal mouse strains tested except, for unknown reasons, in mice carrying the MHC Class‐II molecule H‐2A^b^ (or a gene encoded close to A^b^).^31^ IgE does not enhance in nude mice.^30^

#### 2.2.1 Fc receptors and IgE‐mediated enhancement

IgE‐mediated enhancement is abrogated in CD23 KO mice^33^, ^35^, ^37^, ^41^, ^42^ but normal in mice lacking the other IgE‐binding receptors: FcεRI+FcγRIII+FcγRIV (FcRγ‐chain KO)^43^ or FcγRIIB.^44^ Thus, CD23 is the only IgE‐binding receptor required for IgE‐mediated feedback enhancement. This receptor is constitutively expressed on B cells and FDC (follicular dendritic cells) in mice, and experiments in bone marrow chimeras showed that expression on B cells, but not on FDC, are required for IgE‐mediated enhancement of antibody and T‐cell responses.^33^, ^35^ Enhanced positive signaling via co‐crosslinking of BCR and CD23 on the B cells is unlikely because transfer of CD23^+^ B cells to CD23 KO mice rescued the ability of CD23‐negative B cells to respond to IgE–antigen.^35^ Thus, when CD23^+^ B cells are present in an animal, also CD23‐negative B cells are stimulated to antibody production. IgE enhances the antibody response equally well in IL‐4 KO and wild‐type mice although the former have a fivefold lower expression of CD23 on B cells.^39^ Enhancement is normal in transgenic mice, overexpressing CD23 on their B cells.^29^ These observations suggest that the expression level of CD23 is not critical for the enhancing effect but that constitutive expression suffices.

#### 2.2.2 Complement and IgE‐mediated enhancement

IgE does not activate complement, but human CD21 (CR2) and CD23 are a ligand–receptor pair.^45^ However, involvement of CR2 in IgE‐mediated enhancement is unlikely because IgE enhances both antibody and T‐cell responses normally in mice lacking CR1 and CR2.^34^, ^46^

#### 2.2.3 Mechanism behind IgE‐mediated enhancement

The ability of IgE to enhance the in vivo antibody^22^, ^33^, ^37^ and CD4^+^ T‐cell^29^, ^35^, ^36^ responses, and the requirement for CD23^+^ B cells,^33^, ^35^ was completely in line with the idea that IgE operated by increasing B‐cell‐mediated presentation of IgE‐complexed antigen to T cells, shown to take place in vitro. However, a subsequent observation challenged this hypothesis: OVA‐TNP administered intravenously to mice together with TNP‐specific IgE was captured by CD23^+^ B cells in the blood and transported to splenic follicles where it could be detected on follicular B cells after 30 min.^36^ This revealed a hitherto unknown role for CD23^+^ B cells and led to the conclusion that they either play a dual role, both transporting and presenting IgE–antigen complexes, or that they operate only as transporters. Analysis of whether B cells or dendritic cells from mice immunized with IgE and antigen activated specific CD4^+^ T cells showed that CD11c^+^ cells (subsequently identified as conventional CD8α^−^ dendritic cells^38^), and not CD23^+^ B cells, were the antigen‐presenting cells.^40^

Based on these observations it appears that the role of CD23^+^ B cells in the chain of events leading to IgE‐mediated enhancement of antibody responses is to transport the immune complexes to splenic B‐cell follicles. The antigen is then transferred to dendritic cells which present it to CD4^+^ T cells. Activated specific CD4^+^ T cells will provide efficient help to cognate B cells, resulting in the enhanced formation of germinal centers and antibody responses observed. It is not known how the antigen is delivered from CD23^+^ B cells to dendritic cells, but the mere presence of dendritic cells in follicles and at the T‐/B‐cell border would allow close interactions.^47^ Moreover, specific CD4^+^ T cells localize to the border of the T cell zone, near B‐cell follicles, 12 h after immunization with IgE‐antigen,^36^ and CD8α^−^ conventional dendritic cells migrate from the marginal zone bridging channel into the T‐cell zone a few hours after immunization.^38^ The simplest explanation for how dendritic cells acquire IgE antigen would be that the high antigen concentration in B‐cell follicles, following the active transport of IgE–antigen, triggers neighboring dendritic cells to internalize antigen via default mechanisms. Another possibility is that IgE–antigen is processed by B cells, released as B‐cell exosomes and subsequently internalized by dendritic cells.^42^ Murine dendritic cells do not express CD23,^38^ excluding uptake of antigen by CD23‐mediated endocytosis.

#### 2.2.4 Summary and outlook

**FIGURE 1:**
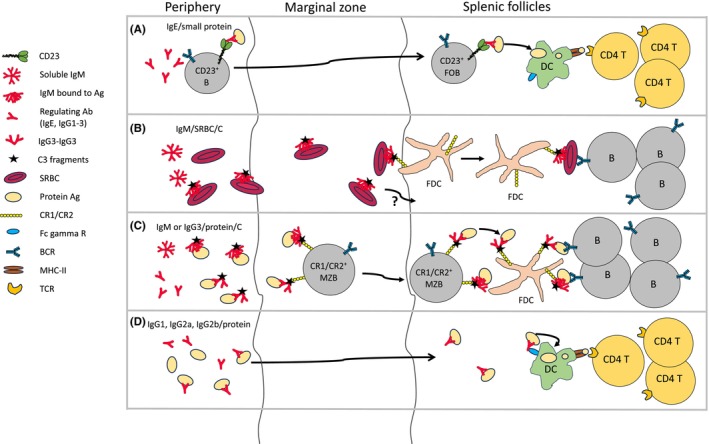
Suggested mechanisms behind antibody feedback enhancement. (A) IgE in complex with small protein antigens is captured in the blood by recirculating B cells expressing the low‐affinity receptor for IgE (CD23) and transported to follicles where it is found on follicular B cells (FOB). The increased concentration of antigen in the follicles facilitates antigen uptake by conventional dendritic cells (DC) and presentation to specific CD4^+^ T cells. These become activated and proliferate and provide efficient help to cognate B cells leading to the enhanced antibody production. (B) IgM in complex with sheep erythrocytes (SRBC) activate complement (C), is concentrated on follicular dendritic cells (FDC) and activate B cells in a similar way as IgM– and IgG–protein immune complexes (see C). It is not known how the transport of IgM‐SRBC from MZ to follicles takes place. Hypothetically, FDC may capture these immune complexes directly from the MZ. (C) IgM or IgG3 in complex with protein antigens activate complement leading to deposition of C3 fragments on the immune complexes. These are transported from the marginal zone (MZ) to follicles by CR1/CR2^+^ MZ B cells (MZB) and transferred to FDC which present the antigen to B cells. The focusing of antigen on FDC will lead to enhanced B‐cell activation and antibody production. (D) IgG1, IgG2a, and IgG2b in complex with protein antigens are endocytosed by DC via activating FcγRs and presented to CD4^+^ T cells. These become activated, proliferate and provide help to cognate B cells, resulting in enhanced antibody production.

The mechanism behind IgE‐mediated feedback enhancement can be described as a relay with antigen as the baton: IgE–antigen is captured by CD23^+^ B cells in the blood, transported to follicles, taken up by dendritic cells, and presented to CD4^+^ T cells (Figure 1A).

Whether IgE‐mediated enhancement of antibody and T‐cell responses plays a physiological role is an open question. Normal serum levels of IgE are very low, approximately 100 ng/mL blood in humans and presumably similar in mice. The lowest IgE concentration found to enhance an antibody response is 10 μg/mouse, that is, ≈5000 ng/mL blood.^22^, ^30^ IgE increases in allergies and high levels may be present locally, but whether CD23 plays a role in allergies is uncertain. IgE concentrations are also high during parasite infections and increased levels of specific IgE have been reported during *Puumala virus* infections.^48^ Because IgE levels are rarely determined during infections, it cannot be excluded that additional pathogens induce IgE responses. Circulating human B cells are preloaded with IgE bound to CD23, thus reflecting the immune history of the individual, and may provide binding opportunities also when serum IgE is low.^49^ Transport of pathogen antigens, captured by IgE, to follicles by CD23^+^ B cells may play a role in rapid induction of antibody responses and constitute an alternative route to the complement dependent transport of other types of immune complexes (Section 2.3). CD23 expressed on human lung epithelial cells^50^ as well as rat^51^ and human^52^ intestinal cells can mediate transcytosis of IgE–antigen complexes, which is in line with the observation that CD23^+^ B cells act as transporters of IgE–immune complexes. CD23 is an unusual Fc receptor, belonging to the C‐type lectin family. Receptors of this family often function as pattern recognition receptors^53^ and it is an intriguing possibility that CD23 would bind directly to certain pathogens without the requirement for IgE, thus providing rapid transport of blood‐borne pathogens to the spleen without prior immunization.

Many other effector functions than the ability to transport IgE–antigen and to enhance in vivo antibody responses have been described for membrane‐bound as well as soluble CD23.^54^ However, these mechanisms often seem to be independent of IgE‐binding to CD23 and are separate from the transportation function by CD23 expressed on B cells described here.

### 2.3 Enhancement by IgM

The most well‐known feedback effect of IgM is to enhance antibody responses. This function was clearly established by Henry and Jerne, who showed that passively administered specific IgM (named 19S at the time) enhances and IgG (7S) suppresses antibody responses to SRBC.^55^ When a mixture of the two was administered, the response was intermediate and could be mathematically predicted.^55^ However, high doses of IgM as well as administration of IgM after the antigen may lead to suppression (Section 3.2).

IgM‐mediated enhancement has been observed against large antigens such as erythrocytes,^34^, ^55^, ^56^, ^57^, ^58^, ^59^, ^60^, ^61^, ^62^, ^63^, ^64^, ^65^ KLH,^21^, ^64^, ^66^, ^67^ and malaria parasites,^68^ while enhancement of responses to small proteins like BSA and OVA has not been reported. In order to enhance, IgM must be administered together with suboptimal doses of antigen^55^, ^69^, ^70^, ^71^ and within a few hours of the antigen.^56^, ^69^, ^70^ IgM is effective over a wide dose range^57^, ^59^ but may turn suppressive with increasing doses.^72^, ^73^ The number of single B cells producing IgM anti‐SRBC are increased after 3 days^57^, ^59^, ^74^ and the ensuing IgG anti‐SRBC response is sustained for at least 3 months.^62^ In the majority of experiments, IgM is administered intravenously, but also intraperitoneal administration works well. IgM‐mediated enhancement is antigen but not epitope specific, that is, IgM specific for one epitope will enhance also responses to other epitopes on the same antigen.^21^, ^55^, ^56^, ^59^, ^60^, ^64^, ^68^, ^71^, ^75^

IgM enhances primary IgM^34^, ^55^, ^56^, ^57^, ^58^, ^59^, ^60^, ^61^, ^62^, ^63^, ^64^, ^65^ and IgG responses,^34^, ^59^, ^62^, ^63^, ^64^ including all IgG subclasses,^61^ IgE,^76^ priming for secondary antibody responses,^62^, ^67^ and development of germinal centers.^64^, ^67^, ^77^ IgM has been shown to regulate B‐cell selection in germinal centers by masking antigenic epitopes.^78^ Investigations on IgM‐effects on T cells are rare. A monoclonal IgM anti‐malaria antibody enhanced T‐cell responses, as measured by its ability to prime for IgG anti‐hapten responses.^68^ On the other hand, IgM had no effect on primary T‐cell responses in mice adoptively transferred with OVA‐specific transgenic CD4^+^ DO11.10 T cells.^64^

The majority of studies have been performed in mice and IgM enhances in all wild‐type strains tested, except old MRL/lpr mice, probably owing to a disrupted splenic micro‐anatomy.^79^ IgM does not enhance in nude mice.^60^, ^71^, ^80^ IgM‐mediated enhancement has been demonstrated in humans: during studies of RhD prophylaxis, IgM anti‐RhD enhanced and IgG anti‐RhD suppressed the anti‐RhD response.^4^

#### 2.3.1 Fc receptors and IgM‐mediated enhancement

There are several Fc receptors for IgM,^81^ but their role in IgM‐mediated enhancement has not been addressed. Involvement of FcμR (TOSO/FAIM3), selectively binding IgM and expressed on B cells in mice, is however unlikely: a mutant IgM antibody which bound normally to FcμR was unable to enhance antibody responses (owing to its ability to activate complement).^64^

#### 2.3.2 Complement and IgM‐mediated enhancement

IgM is a very efficient activator of complement and this feature turned out to be crucial for its enhancing effect. Monoclonal TNP‐specific IgM (IgM^#13^) with a point mutation (serine at position 436 substituted with proline; ser436pro) in the third constant domain of the μ heavy chain proved to be unable to activate complement^82^ and to enhance antibody responses to SRBC‐TNP.^75^ Likewise, polyclonal IgM anti‐SRBC, derived from the Cμ13 knock‐in mouse strain with the same ser436pro mutation, was unable to enhance.^64^ Monomeric IgM, which is unable to activate complement, cannot enhance,^67^ and IgM does not enhance in mice lacking CR1/CR2,^34^, ^63^ C1q,^65^ or C3.^65^

In an attempt to elucidate by which mechanisms complement activation was important for IgM‐mediated enhancement, we tested whether CR1/CR2 were involved. In mice, these two receptors are splice variants encoded by the Cr2 gene. They are expressed on B cells and FDC^83^ and bind C3 split products and can therefore capture opsonized IgM‐antigen complexes. Initial experiments in mice in which CR1/CR2 were blocked with monoclonal antibodies showed dramatic effects. Antibody responses not only to IgM‐SRBC but also to SRBC and HRBC, administered alone as controls, were severely impaired. Likewise, antibody responses to HRBC and KLH, administered without IgM, were impaired.^84^ These results suggested a more general role for CR1/CR2 than to cause IgM‐mediated enhancement. When Cr2 KO mice became available, the crucial role of these receptors in antibody responses to uncomplexed antigen was further established.^85^, ^86^

The antibody response in Cr2 KO mice is characterized by impaired primary and secondary antibody responses, with a more severe impairment of IgG than IgM responses. Moreover, antibody responses can be rescued by immunization with high antigen doses or by the use of adjuvants. These features resemble those found in mice lacking C1q, C4, or C3 (reviewed in^13^) and implies a linear relationship where complement activation via the classical pathway, initiated by C1q, leads to generation of C3 split products and ligation of CR1/CR2.

#### 2.3.3 A paradox: Classical pathway complement activation is crucial for primary antibody responses

Whereas lack of classical pathway factors severely impaired antibody responses, lack of factors from the alternative or lectin pathway had no, or minor, effects.^13^ The dependence of classical pathway complement activation for primary antibody responses constituted a paradox because antibodies in complex with antigen are the most efficient activators of this pathway. This raises the question of how the classical pathway is activated in naïve mice when only very little specific IgM (and other isotypes) is available. A possible explanation was suggested by experiments in mice lacking secretory IgM.^87^ These animals had an impaired primary antibody response which could be rescued by transfer of IgM from normal serum. Therefore it appeared logical that natural IgM, present already at the time of immunization, would bind antigen, activate C1q and facilitate early antibody responses. To test this hypothesis, a knock‐in mouse strain (Cμ13) with the ser436pro point mutation was constructed.^88^ In these mice all IgM antibodies, including preexisting natural IgM, will carry the mutation and be unable to bind C1q. Unexpectedly, the antibody responses to SRBC and KLH in Cμ13 mice were usually indistinguishable from responses in wild‐type mice.^88^, ^89^ Occasionally responses were lower, but never as severely impaired as in C1q, Cr2, or C3 KO mice.^88^, ^89^ Therefore, complement activation by natural, preexisting IgM cannot explain the crucial importance of classical pathway activation in antibody responses, at least not to SRBC or KLH. The question of how C1q is involved in primary antibody responses to uncomplexed antigens remains to be answered. Does complement activation by other natural, preexisting antibodies, such as IgG3, play a role? Is the lectin pathway involved? Are non‐immunoglobulin activators of the classical pathway involved? Notably, antibody responses were normal in the absence of CRP (C‐reactive protein), SAP (serum amyloid component), or SIGN‐R1 (specific intracellular adhesion molecule‐grabbing non‐integrin R1), all of which are able to activate the classical pathway.^88^

#### 2.3.4 Mechanism behind IgM‐mediated enhancement

In mice, CR1/CR2 are expressed on B cells and FDC,^83^ both involved in the generation of antibody responses. Two mutually not exclusive hypotheses for how IgM via complement enhances antibody responses are currently discussed. Co‐crosslinking of BCR and the CR2/CD19/CD81 co‐receptor complex lowers the threshold for B‐cell activation in vitro,^90^, ^91^, ^92^ and may be initiated in vivo by opsonized IgM immune complexes. Alternatively, IgM immune complexes could be transported to B‐cell follicles, captured by FDC and presented to B cells. Opsonization of IgM–SRBC complexes in blood is rapid. When SRBC are administered intravenously to a mouse which has previously received specific IgM, C3 split products are deposited on the SRBC within 1 min.^13^, ^64^ Endogenous IgM from wildtype, but not Cμ13 mice, also causes rapid deposition of C3 after a secondary immunization with SRBC.^89^

Studies in bone marrow chimeric mice showed that IgM‐mediated enhancement was mainly dependent on expression of CR1/CR2 on FDC although a weak response occurred when these receptors were expressed on B cells only.^63^ These observations indicate that FDC are important players in IgM‐mediated enhancement and are in line with the findings that monoclonal IgM anti‐NP (4‐hydroxy‐3‐nitrophenylacetyl) increased deposition of NP‐KLH on FDC in splenic follicles, provided C3 and CR1/CR2 were present.^67^ In complement deficient mice antigen remained in the MZ (marginal zone).^67^ The transport of these IgM‐immune complexes from the MZ to FDC was performed by MZ B cells,^77^ which express high levels of CR1/CR2. It is now known that MZ B cells constantly shuttle between the MZ and follicles, whether they carry any “cargo” or not.^93^, ^94^

SRBC are more difficult to detect immunohistochemically than proteins, perhaps owing to the fragile nature of erythrocytes. Early studies with ^51^Cr labeled SRBC showed that the splenic concentration of SRBC increased in parallel with antibody responses after co‐administration of IgM.^57^ IgM‐mediated enhancement of antibody responses to SRBC worked well in chimeric mice in which the B cells, including MZ B cells, did not express CR1/CR2.^63^ Thus, unlike NP‐KLH,^77^ SRBC are not transported by MZ B cells, at least not bound to CR1/CR2. Involvement of FcμR, also expressed on MZ B cells, is unlikely because mutant IgM which had lost its ability to activate complement, but bound normally to FcμR, was unable to enhance antibody responses.^64^ It is presently not understood how SRBC reach the follicles. As IgM‐mediated enhancement of SRBC responses is heavily dependent on CR1/CR2‐expressing FDC,^63^ one way to explain how SRBC end up in follicles would be that FDC extend their processes into the MZ to directly capture opsonized IgM–SRBC complexes. Conventional dendritic cells are known to extend processes across the epithelial layer of the gut to sample antigen^95^ and subcapsular sinus macrophages extend protrusions through the subcapsular sinus wall in lymph node follicles and deliver antigen to B cells.^96^

#### 2.3.5 Summary and outlook

Enhancement of antibody responses by specific IgM is dependent on the ability of IgM to activate complement. The most likely mechanism is increased follicular localization of antigen on FDC expressing CR1/CR2, thus increasing the effective antigen concentration in an area crucial for the generation of antibody responses (Figure 1B,C). Lowering the threshold for B‐cell activation by co‐crosslinking BCR and the CR2/CD19/CD81 co‐receptor complex may play an additive role. The requirement for classical pathway complement activation in generation of early primary antibody responses to SRBC, KLH, or NP‐KLH, administered alone, cannot be explained by activation via natural IgM.^88^, ^89^ This paradox is still in search of an explanation.

IgM is the first antibody class to respond after immunization and it appears logical that it plays a role in augmenting early immune responses. The ability of IgM to enhance responses to low antigen doses may be important at the onset of an infection when only low doses of the pathogen are present. Feedback regulation by IgM has primarily been studied in settings with passive administration of preformed specific IgM, but also endogenously produced IgM can enhance late primary and secondary IgG responses.^89^

### 2.4 Enhancement by IgG3

IgG3 is a subclass with unusual features and was the last antibody isotype found to feedback‐enhance antibody responses.^97^ It is the predominant IgG subclass responding to T cell independent antigens of Type 2 and constitutes only a small fraction of the response to T cell dependent antigens. IgG3 plays an important role in the defense against bacterial infections.^98^

Monoclonal IgG3 anti‐TNP, administered with BSA‐TNP or OVA‐TNP, enhances primary antibody responses to the carrier proteins,^97^, ^99^, ^100^, ^101^ priming for secondary antibody responses,^101^ and development of germinal centers.^101^ Little or no effect on T helper cell priming is observed.^99^

#### 2.4.1 Complement and IgG3‐mediated enhancement

IgG3 activates complement both via the alternative and classical pathway^102^ and has the ability of intermolecular cooperativity.^103^ This means that when one IgG3 molecule has bound to a surface, it attracts other IgG3 molecules to interact non‐covalently via their Fc regions, presumably facilitating C1q binding because a single IgG molecule has too low affinity to bind C1q. In contrast, other murine IgG subclasses have to rely on at least two molecules by chance ending up close enough to cooperate in C1q‐binding, thus requiring high concentrations of specific IgG in serum. This difference may explain why IgG3 relies on complement while IgG1, IgG2a, and IgG2b primarily relies on FcγRs to exert feedback regulation (Section 2.5). IgG3‐mediated enhancement of primary IgG responses was severely impaired in the absence of CR1/CR2,^97^, ^100^ C3,^97^, ^100^ or C1q,^101^ and induction of germinal centers required CR1/CR2.^100^

#### 2.4.2 Fc receptors and IgG3‐mediated enhancement

IgG3 was for a long time a subclass in search of an FcR, but it is now known that it binds to FcγRI.^104^ Enhancement of primary antibody responses was normal in FcRγ‐chain KO mice^97^ (lacking FcγRI, FcγRIII, and FcγRIV) as well as in mice selectively lacking FcγRI.^99^

#### 2.4.3 Mechanism behind IgG3‐mediated enhancement

In mice immunized intravenously with monoclonal IgG3 anti‐TNP and OVA‐TNP, antigen could be detected on MZ B cells and FDC in the spleens after 2 h.^100^ No antigen was observed in follicles of Cr2 KO mice or in mice immunized with OVA‐TNP alone. Inhibition of the shuttling of MZ B cells between the MZ and the follicle abolished IgG3‐mediated follicular localization of the antigen.^100^ Maximal IgG3‐mediated enhancement required CR1/CR2‐expression on both FDC and B cells,^100^ resembling the findings in mice immunized with IgM and KLH‐NP or BSA‐NP.^77^ A likely explanation for IgG3‐mediated enhancement is that MZ B cells transport the opsonized immune complexes from the MZ to B‐cell follicles where it is captured by FDC and presented to B cells. In analogy with IgM‐mediated enhancement, the increased concentration of antigen in follicles will activate the immune system just as efficiently as a higher dose of antigen alone would do, thus explaining the enhanced antibody and germinal center responses.

#### 2.4.4 Summary and outlook

The *modus operandi* of IgG3 in feedback regulation has many similarities to that of IgM. Both rely on complement rather than on FcRs, both are poor inducers of T helper cells and probably operate via efficient presentation of antigen on FDC to B cells (Figure 1C). One single IgM molecule suffices to bind C1q, and IgG3's capacity of cooperative binding may lead to the same result because it requires only one IgG3 molecule for the initial binding. The rapid complement activation may facilitate early feedback effects as well as defenses against infections early in an immune response. A difference between IgM and IgG3 in feedback regulation is the type of antigen they can enhance responses against. IgM requires a large antigen whereas IgG3 works well with BSA and OVA. Presumably, IgM would need an antigen large enough to allow binding of several of its antigen binding sites to induce the conformation change required to expose C1q binding sites. On the other hand, perhaps a single IgG3 molecule bound to an antigen may capture other IgG3 molecules via Fc–Fc interactions, thereby allowing C1q binding.

### 2.5 Enhancement by IgG1‐, IgG2a, and IgG2b

Feedback regulation by IgG is a complex subject. There are several IgG subclasses which can act via complement and/or Fc receptors. FcγRs can be either activating or inhibitory and are expressed, and often co‐expressed, on many different cell types and the efficiency in complement activation varies with subclass.

IgG‐mediated enhancement has mainly been described for protein antigens. However, enhancement of antibody responses to SRBC has been observed,^57^, ^105^, ^106^, ^107^, ^108^, ^109^ although IgG usually suppresses SRBC responses (Section 3.3). Generally, low concentrations, low affinity, and low epitope density predispose IgG to enhance rather than suppress SRBC responses.

Monoclonal hapten‐specific IgG1, IgG2a, and IgG2b antibodies enhance primary IgM and IgG responses against haptenated KLH, OVA, BSA, and tetanus toxoid.^21^, ^41^, ^43^, ^66^, ^110^, ^111^, ^112^, ^113^, ^114^ The enhancement can be several 100‐fold^21^ and serum titers remain high for weeks.^21^, ^32^, ^111^ There is no apparent correlation between affinity and ability to enhance.^21^, ^110^ Monoclonal IgG2a or IgG2b also enhance secondary responses^20^, ^21^ and IgG2a enhances priming for memory responses.^113^ Proliferation of OVA‐specific CD4^+^ T cells was enhanced after administration of IgG2a anti‐TNP and OVA‐TNP^44^ and IgG1 increased the frequency of somatic mutations in germinal centers.^115^

#### 2.5.1 Complement and IgG1‐, IgG2a‐, and IgG2b‐mediated enhancement

Enhancement by IgG1, IgG2a, and IgG2b can take place without involvement of complement as shown in experiments where monoclonal TNP‐specific IgG antibodies were administered intravenously together with KLH‐TNP or BSA‐TNP. Monoclonal IgG2a, which had lost its ability to bind C1q owing to a point mutation, was as efficient as the wild‐type antibody.^111^ Likewise, a monoclonal IgG1 antibody, inherently unable to activate complement, enhanced efficiently.^111^ IgG2b could enhance primary antibody responses in C3‐depleted mice^111^ and IgG2a enhanced in Cr2 KO mice.^34^ The latter observation is interesting because it shows that immune complexes containing IgG2a (or IgE), unlike immune complexes containing IgM or IgG3, can escape the requirement of CR1/CR2 in the generation of antibody responses.^34^, ^63^, ^97^, ^100^

Although enhancement by IgG1, IgG2a, and IgG2b can take place without involving complement, the mere fact that IgG2a and IgG2b are able to activate complement suggest that they would utilize this in feedback enhancement during the “right” conditions. Depletion of C3 prevented follicular trapping of aggregated human IgG^116^ and complexes of polyclonal IgG‐KLH‐DNP,^117^ and the ability of polyclonal IgG‐KLH‐DNP to prime B cells was impaired.^117^ These two antigens are large and, as opposed to small proteins like OVA and BSA, are likely to allow binding of several IgG molecules and facilitate capture of C1q.

#### 2.5.2 Fc receptors and IgG1‐, IgG2a‐, and IgG2b‐mediated enhancement

IgG1, IgG2a, and IgG2b have severely impaired capacity to enhance primary IgG responses in FcRγ‐chain KO mice, lacking FcγRI, FcγRIII, and FcγRIV.^43^, ^44^, ^113^ Moreover, the ability of IgG2a to prime for memory responses^113^ and to enhance proliferation of specific CD4^+^ T cells^44^ was abolished. In mice selectively lacking FcγRIII, IgG2a and IgG2b enhanced normally whereas IgG1‐mediated enhancement was partially impaired.^43^ On the other hand, an “enhanced enhancement” was seen in mice lacking the inhibitory FcγRIIB.^43^, ^44^ For example, after immunization with IgG2a anti‐TNP and BSA‐TNP, the IgG anti‐BSA response in wild‐type mice was 10‐fold higher than in controls given BSA‐TNP alone but in FcγRIIB KO mice enhancement was 267‐fold. This reveals the remarkable inhibitory effect of FcγRIIB on antibody responses when it is present, that is, in wild‐type mice.^43^ IgG2a‐mediated enhancement required expression of activating FcγRs on a bone marrow‐derived cell type,^113^ thus excluding FDC which are not derived from the bone marrow. More recent data show that IgG‐mediated enhancement of T helper cell responses require dendritic cells.^118^

#### 2.5.3 Mechanism behind IgG1‐, IgG2a, and IgG2b‐mediated enhancement

The most likely mechanism behind feedback enhancement by IgG1, IgG2a, and IgG2b is FcγR‐mediated endocytosis of IgG–antigen complexes by dendritic cells followed by enhanced proliferation of T helper cells and subsequent efficient help to cognate B cells (Figure 1D). This resembles the suggested mechanism for IgE‐mediated enhancement, but without the initial capture and transport of IgE–antigen complexes by circulating CD23^+^ B cells to follicles.^36^ B cells express FcγRIIB, and can bind IgG–antigen in the circulation,^41^ but since IgG‐mediated enhancement is enhanced in FcγRIIB KO mice this may not be relevant.^43^, ^44^ Details on how and where IgG–antigen complexes reach dendritic cells are not known. However, at least part of the interaction probably takes place in the spleen because splenic CD4^+^ OVA‐specific T cells proliferate vigorously 3 days after administration of IgG2a anti‐TNP and OVA‐TNP.^44^ During conditions facilitating complement activation, such as large/aggregated antigens and high IgG concentrations, it is possible that IgG2a and IgG2b, similarly to IgG3 and IgM, increase antigen localization to follicles. This would play an additive role to the FcγR‐dependent antigen presentation pathway in IgG‐mediated feedback enhancement.

## 3 ANTIBODY FEEDBACK SUPPRESSION

IgG is the most well‐studied suppressive isotype and presumably the most important one owing to its high serum concentration, high affinity, and long half‐life. However, both IgM and IgE are able to suppress antibody responses.

### 3.1 Suppression by IgE

Monoclonal TNP‐specific IgE antibodies suppressed the response to SRBC when administered with SRBC‐TNP.^119^ Suppression took place in the absence of the known receptors for IgE, FcεRI, CD23, FcγRIII, FcγRIV, or FcγRIIB.^120^

### 3.2 Suppression by IgM

IgM is best known for its ability to enhance antibody responses. However, IgM administered 1 or 2 days after, instead of before, SRBC became suppressive and IgM suppressed antibody responses in vitro.^56^ Low doses of IgM enhanced while high doses suppressed SRBC responses.^72^, ^73^

### 3.3 Suppression by IgG

IgG suppresses antibody responses to erythrocytes, viruses, and proteins administered in adjuvants (reviewed in^11^, ^14^). The majority of our knowledge about IgG‐mediated suppression comes from studies of mice immunized with SRBC or haptenated SRBC.^55^, ^107^, ^121^, ^122^ Recently, suppression of responses to murine erythrocytes expressing transgenic protein epitopes (e.g., hen egg lysozyme, OVA, and human blood group proteins)^123^ as well as to viral proteins^5^, ^6^, ^7^, ^16^, ^17^, ^18^, ^19^ and proteins from malaria parasites^5^ have been studied.

Passively administered IgG frequently suppresses more than 99% of primary IgM responses, often measured as single splenic B cells by hemolytic plaque assays.^55^, ^119^, ^121^, ^124^ Serum IgG responses,^122^, ^125^ splenic germinal center B cells,^122^ extrafollicular antibody secreting cells,^122^ and IgG‐producing long‐lived plasma cells in the bone marrow^122^ are also suppressed. Induction of memory and secondary antibody responses are suppressed although usually not as completely as primary responses.^5^, ^62^, ^124^ However, the relative suppression of primary antibody responses and priming for memory was similar in mice primed with IgG and SRBC and boosted with SRBC 70 days later, indicating that plasma cells and memory B cells can be equally efficiently suppressed at priming.^122^ Interestingly, IgG does not significantly suppress T helper cell responses to SRBC^119^, ^126^, ^127^ or to viruses.^9^, ^128^

High doses of IgG are more suppressive than low doses and responses to high doses of antigen are more difficult to suppress than responses to low doses. High‐affinity antibodies suppress better than low‐affinity antibodies^129^, ^130^ and all murine IgG subclasses can suppress.^110^, ^121^, ^129^ Unlike feedback enhancement, which requires administration of antibody and antigen within hours of one another, IgG administered up to 5 days after SRBC can terminate ongoing antibody responses to SRBC^55^, ^124^, ^131^ or diphtheria toxin,^132^ suggesting that continuous interaction between antigen and B cells is required to sustain a primary antibody response.

#### 3.3.1 Endogenous feedback regulation

Antibody feedback regulation has mainly been studied after passive administration of specific antibodies and antigen to naïve mice. This experimental design does not fully reflect the antibody feedback regulation taking place by endogenous antibodies produced after primary immunization and persisting at the time of a secondary immunization. The net outcome of a recall antibody response will be determined not only by the amount, isotypes, and affinity of the antibodies generated in the primary response, but also by the presence of primed T cells, plasma cells, and memory B cells. Early studies demonstrated the effect of endogenous feedback suppression when removal of specific antibodies from rabbits via exchange transfusion led to an increase in the levels of specific antibodies, resembling a booster immunization.^133^ In line with this, removal of B cells producing antibodies suppressing the response to the RBD‐W determinant on SARS‐CoV‐2 resulted in an increase in plasmablasts with this specificity.^6^ Mice primed with IgM and SRBC, leading to enhanced levels of primary anti‐SRBC antibodies, had lower recall responses than mice primed with SRBC alone. This apparent paradox was explained by endogenous IgG‐mediated suppression: when boosting was delayed until IgG levels had subsided, or when memory cell generation was studied separately in adoptive transfer systems, the recall response in IgM‐SRBC‐primed mice was higher than in controls.^62^ Thus, in spite of more efficient cellular memory, the net serum antibody response can be impaired owing to feedback suppression.

Recently, inhibitory effects of antibodies persisting from previous immunizations with HIV, SARS‐CoV‐2, *Plasmodium falciparum*, or influenza PR8 in mice and/or humans, have been studied in detail.^5^, ^6^, ^7^, ^16^, ^17^, ^18^, ^19^ Specific antibodies suppressed not only serum antibodies but inhibited germinal center B cells, recruitment of naïve B cells to secondary germinal centers, memory B cells, and plasmablasts. Blocking of a specific epitope by IgG often led to responses to other, less immunodominant, epitopes. This implies that feedback suppression drives diversification of antibody responses and provides a mechanism promoting epitope spreading and counteracting the “original antigenic sin” effect. The net effect of endogenous feedback regulation is usually suppression of the response to the epitope recognized. However, also positive endogenous feedback regulation exists. For example, low‐affinity IgG enhanced recall responses to SARS‐CoV‐2 whereas high‐affinity antibodies induced suppression^17^ and IgM generated during a primary response enhanced the response to a booster dose of SRBC.^89^

#### 3.3.2 Fc dependence or not

To understand the mechanism behind IgG‐mediated suppression it is important to determine whether the IgG(Fc) portion is required. The most straightforward approach is to test the suppressive ability of F(ab′)_2_ fragments, but such experiments have given discrepant results. Some investigators find that F(ab′)_2_ are suppressive and others that they are not.^14^ The reason for this discrepancy is not known, but a potential problem is that FcRn (the neonatal FcR) cannot protect F(ab′)_2_ fragments from degradation and that they therefore are eliminated from the circulation much quicker than intact IgG, resulting in a lower effective serum concentration.

Another strategy to determine the importance of the IgG(Fc) portion has been to study whether suppression is epitope specific or not. Epitope specificity of suppression, that is, that IgG only suppresses the response to the epitope it binds, would indicate Fc independence whereas non‐epitope‐specific suppression against all epitopes on the antigen, would indicate Fc dependence. However, both epitope‐specific^107^, ^122^, ^126^, ^134^, ^135^, ^136^ and non‐epitope‐specific^66^, ^107^, ^119^, ^121^, ^126^, ^129^, ^137^, ^138^, ^139^ suppression have been observed.

#### 3.3.3 Fc receptors and IgG‐mediated suppression

An alternative way to determine whether IgG‐mediated suppression is Fc‐dependent or not presented itself when FcγR KO mice became available. We unexpectedly found that IgG could suppress equally well in mice lacking FcγRIIB, all activating FcγRs, or FcRn as in wild‐type mice.^119^ These observations were confirmed and extended and it is now established that suppression is unperturbed in mice lacking the inhibitory FcγRIIB,^119^, ^124^, ^125^, ^140^ the activating FcγRs I + III + IV (FcRγ‐chain KO),^119^, ^122^, ^125^, ^140^, ^141^ both activating and inhibitory FcγRs (FcγRIIB × FcRγ‐chain double KO),^119^ or FcRn (β2‐microglobulin KO).^119^ Suppression of the response to SRBC was normal in (C3 × FcRγ‐chain) double KO mice.^142^ In contrast, IgG did not suppress the response to murine transgenic erythrocytes in these double KO mice, although suppression in the single KO's was unperturbed.^141^

#### 3.3.4 Mechanism(s) behind IgG‐mediated suppression

Several mechanisms have been proposed to explain antibody feedback suppression. They will be discussed below, assuming that a single mechanism plays a major role, although it cannot be excluded that more than one mechanism contributes. A table summarizing the pros and cons for the various hypotheses has been published in a previous review.^14^

##### Epitope masking

The early straightforward explanation to feedback suppression was that antibodies binding to their specific epitope would prevent specific B cells from binding to the same epitope.^10^ This so called epitope masking hypothesis was challenged by data showing that F(ab′)_2_ did not suppress, that non‐epitope‐specific suppression existed, and that co‐crosslinking of the inhibitory FcγRIIB and BCR in vitro inhibited B‐cell activation, all pointing to an Fc‐dependent mechanism. Our initial demonstration that IgG‐mediated suppression was unperturbed in mice lacking FcγRs^119^ was a tipping point in my own thinking about the mechanism. A critical review of the literature made us arrive at the conclusion that most available data were compatible with epitope masking being the major mechanism.^119^, ^143^ At the time, the most favored hypothesis to explain IgG‐mediated suppression was central B‐cell inhibition by co‐crosslinking of BCR and the negatively regulating FcγRIIB, shown to occur in vitro.^144^ The finding that suppression worked well in mice lacking FcγRs, including FcγRIIB,^119^, ^124^ was surprising and created some debate.^145^, ^146^ The data from FcγR KO mice were not easily explained away, but neither were the data from several laboratories, including my own, pointing toward Fc dependence of suppression. In vitro, blocking FcγRIIB+FcγRIII inhibited suppression,^147^ F(ab′)_2_ did not suppress,^148^ and de‐glycosylated IgG (which did not bind FcγRs or activate complement) lost its suppressive effect.^149^ In a direct comparison, IgG did not suppress the in vitro response to SRBC when spleen cells from FcγRIIB KO mice were used, but suppressed efficiently in a parallel in vivo setting.^124^ Thus, in vitro results were not always representative of the in vivo situation.

However, excluding in vitro findings still left unexplained non‐epitope‐specific suppression and inability of F(ab′)_2_ fragments to (sometimes) suppress. The latter observation still awaits an explanation, but the specificity issue can most likely be explained by considering epitope density. In order for IgG to cause non‐epitope‐specific suppression, high epitope density is required (reviewed in^14^). For example, the ability of hapten‐specific IgG antibodies to suppress IgM anti‐SRBC responses increased with the density of TNP or NP conjugated to the SRBC.^107^, ^110^, ^126^ Moreover, an additive suppressive effect of monoclonal antibodies specific for different epitopes on SRBC has been reported.^121^, ^150^ Thus, it is feasible that steric hindrance by IgG, binding to a high‐density epitope, will prevent B cells from binding also to neighboring epitopes, and cause non‐epitope‐specific suppression—without requirement for the IgG(Fc) portion. On the other hand, IgG binding to an epitope expressed at low density would only prevent the B cells specific for this epitope from binding, resulting in epitope‐specific suppression. The ability of IgG to cause non‐epitope specific suppression has mainly been demonstrated for IgM responses and non‐epitope specificity of suppression indeed seems to be easier to induce for IgM‐ than for IgG responses.^107^ In a direct comparison, IgG anti‐NP administered with SRBC‐NP of different NP densities did not suppress the IgG anti‐SRBC response regardless of whether high‐ or low‐NP density SRBC were used.^107^ By contrast, the IgM anti‐SRBC responses were suppressed only when high‐NP density SRBC were used.^107^ The reason for this is not understood, but a possibility is that high‐affinity IgG^+^ B cells compete more efficiently than low‐affinity IgM^+^ B cells with the passively administered IgG for access to neighboring SRBC epitopes.

Thus, excluding in vitro findings, accepting that that non‐epitope‐specific suppression may be caused by steric hindrance, and that F(ab′)_2_ fragments are able to suppress, Fc independence of suppression is compatible with the majority of experimental findings. This suggests that the mechanism behind IgG‐mediated suppression is epitope masking. Recent experiments on endogenous feedback regulation after booster doses of viral antigens or *Plasmodium falciparum* support a major role for epitope masking.^5^, ^6^, ^7^, ^16^, ^17^, ^18^, ^19^

##### Negative regulation of B cells by co‐crosslinking of FcγRIIB and BCR

Inhibition of B‐cell activation via co‐crosslinking of FcγRIIB and BCR, demonstrated in vitro,^144^ cannot be a major mechanism behind IgG‐mediated suppression because it works well in FcγRIIB KO mice.^119^, ^124^, ^125^, ^140^ Moreover, a mutant monoclonal IgG antibody which did not bind FcγRIIB suppressed the expansion of memory B cells as efficiently as the wild‐type antibody^5^ and IgG3 which does not bind to this receptor can suppress.^121^, ^129^ Undoubtedly, FcγRIIB has inhibitory effects in other situations^151^ but its effect may be to modulate rather than to completely suppress antibody responses.

##### Complement‐mediated destruction/elimination of antigen

Complement‐mediated destruction or elimination of antigen has been discussed in feedback suppression of erythrocyte responses. Arguing against a role for complement is that IgE, F(ab′)_2_ fragments as well as monoclonal IgG antibodies, unable to activate complement,^138^ are effective suppressors of anti‐SRBC responses, and that IgG can suppress in mice lacking C1q,^125^ CR1/CR2,^125^ or C3.^125^, ^141^ Involvement of complement would be hard to reconcile with suppression against proteins, which are not susceptible to lysis, and with the observation that IgG administered several days after SRBC can suppress.

##### Clearance

Several observations argue against an exclusive role for clearance in suppression. SRBC are almost completely cleared from the circulation within 1 min of immunization whether IgG is co‐administered or not^126^ but IgG administered several days after SRBC can terminate the antibody response.^55^, ^124^, ^131^ Likewise, IgG administered after completed clearance of malaria sporozoits inhibits induction of B‐cell memory.^5^ Assuming that clearance by IgG is dependent on complement and/or FcγRs, the effective suppression in mice lacking activating FcγRs, complement, or both,^142^ also argues against this mechanism. Moreover, although IgG anti‐SRBC reduced the amount of SRBC in the spleen of wildtype but not FcRγ KO mice, suppression worked equally well in both strains.^122^, ^126^ Epitope‐specific suppression is difficult to encompass with clearance as the entire antigen would be eliminated. Allogeneic erythrocytes persist longer in the blood than xenogeneic SRBC and increased clearance may be more relevant with these antigens. However, there was no correlation between the ability of monoclonal IgG antibodies, specific for transgenic murine erythrocytes, to suppress and their ability to induce clearance^123^, ^139^ or between the suppressive ability of human anti‐RhD and clearance.^152^, ^153^ Although IgG is clearly able to suppress without inducing clearance, it is possible that this mechanism plays a role in certain settings.

##### Trogocytosis (antigen modulation)

A recently suggested mechanism for suppression, based on experiments using allogeneic mouse erythrocytes as antigen, is that the IgG antibodies cause removal of their specific epitope from the erythrocyte surface via trogocytosis.^123^, ^139^ Several explanations for why this would lead to suppression were proposed: erythrocyte damage/death, reduced surface expression of the epitope, or immune deviation resulting in an antibody response against IgG instead of erythrocytes.^123^ The requirements for trogocytosis are still unclear. For example, it is not clear whether Fc receptors are involved, and at present it is difficult to evaluate how well the hypothesis agrees with other experimental observations. Trogocytosis is difficult to reconcile with suppression of antibody responses against viruses and proteins in adjuvants since they do not have cell membranes.

#### 3.3.5 Summary and outlook

To summarize, epitope masking is compatible with a number of observations: (i) efficient suppression in mice lacking FcγRs and/or complement, (ii) suppression by antibodies unable to activate complement or to bind FcγRIIB, (iii) suppression by F(ab′)_2_ fragments, IgM, and IgE, (iv) epitope specificity at low epitope density and non‐epitope specificity at high epitope density, (v) lack of suppression of T helper cell responses (masking of epitopes recognized by B cells would not prevent presentation of other epitopes to T cells), (vi) correlation between suppression and high affinity of IgG, and (vii) suppression without correlation to clearance. Hard to reconcile with epitope masking is the inability of F(ab′)_2_ fragments to suppress, reported in some studies, and the abrogation of suppression in (C3 × FcRγ‐chain) double KO mice. Notably, other studies find that F(ab′)_2_ fragments can suppress and that suppression works in (C3 × FcRγ‐chain) double KO mice. A muddy point in the epitope masking hypothesis is how to explain the long‐term fate of the antigen. Possibly the high‐affinity IgG antibodies simply bind to and hide the antigen until it is destroyed by default mechanisms and rendered non‐immunogenic to B cells. The observation that normal CD4^+^ T‐cell priming is induced, although the antibody response is severely impaired, suggests that fragments of the antigen are available for presentation on MHC Class II although B‐cell epitopes are unavailable.^9^, ^119^, ^126^, ^127^, ^128^

Feedback suppression in a clinical situation can cause problems by interfering with vaccinations, both in infants and adults. On the other hand, suppressing the response to an immunodominant epitope may be beneficial, leading to epitope spreading and a broader pathogen‐specific antibody repertoire. The prevention of alloimmunization of RhD‐negative women carrying RhD‐positive fetuses is a brilliant example of feedback suppression used successfully in the clinic. Currently, anti‐RhD is isolated from pools of human serum and much effort has been put into developing monoclonal IgG anti‐RhD. Such antibodies would eliminate the risk of transferring blood‐borne infections and also solve the problem with the decreasing availability of human anti‐RhD serum owing to successful RhD prophylaxis. Understanding the mechanism behind RhD prophylaxis is crucial to develop efficient monoclonal IgG anti‐RhD.^154^ Suppression of responses to allogeneic RhD^+^ erythrocytes is generally not believed to be caused by epitope masking, a major argument being that the amount of anti‐RhD which suffices to suppresses maternal anti‐RhD responses does not saturate all RhD epitopes.^155^ The antibody response to RhD in humans is much weaker than the response to SRBC in mice, and perhaps partial masking of RhD epitopes will suffice to lower the threshold for alloimmunization? It seems strange that feedback suppression of responses to SRBC and to allogeneic erythrocytes would follow completely different rules, but this enigma awaits a solution.

## 4 CONFOUNDING FACTORS IN STUDIES OF ANTIBODY FEEDBACK REGULATION

The outcome of antibody feedback regulation is determined by many factors, making it challenging to compare experimental outcomes. IgM, all IgG subclasses, and IgE can either enhance or suppress antibody responses. Thus, the direction of feedback regulation is not determined by inherent features of the different isotypes, but by other factors.

In vitro and in vivo studies may give different results as discussed above. For example, IgE–antigen complexes are taken up and presented to CD4^+^ T cells by B cells in vitro, but not in vivo, and IgG suppresses antibody responses to SRBC independently of the Fc portion in vivo, but not in vitro.

Timing between administration of antibody and antigen plays a role. IgM‐, IgG3‐, and IgE‐mediated feedback enhancement is dependent on redistribution of antigen in an optimal way. Therefore antibodies must be present at the time of immunization, or administered within a few hours after the antigen, in order to be effective. By contrast, timing is relatively unimportant for induction of suppression, as IgG administered several days after antigen may terminate a primary response.

The type of antigen and whether epitope‐specific or non‐epitope‐specific antibody responses are studied may give different results. For example, hapten‐specific IgG or IgE enhance carrier responses to BSA‐TNP but may suppress carrier responses to SRBC‐TNP (when the epitope density is high). Whether hapten‐specific responses are affected is not always analyzed. For practical reasons, studies are often set up so that the passively administered antibodies (in this case TNP‐specific) can be distinguished from the actively produced antibodies. Therefore, potential suppression of, for example, TNP responses after administration of BSA‐TNP will go unnoticed. Assuming that epitope masking is an important mechanism behind feedback suppression, the antibody response to the hapten epitopes would always be suppressed, provided the regulating antibody has high enough affinity to outcompete specific B cells and is administered in sufficient quantities. Suppression against the specific epitope to which the regulating antibody binds and concomitant enhancement against nonspecific epitopes on the same antigen has been described.^107^, ^108^, ^109^ Notably, suppression could be induced with F(ab′)_2_ fragments, while enhancement required the IgG(Fc) portion.^109^ This is consistent with the idea that suppression depends on epitope masking, and when enhancement against the unmasked epitopes takes place, it requires complement and/or Fc receptors. The competition between suppressive and enhancing effects of SRBC‐specific IgG and IgM, resulting in an intermediate response, is another illustration of the balance between enhancement and suppression.^55^

The concentration of antibodies in relation to antigen influences regulation.^11^ For example, low doses of IgM enhance while higher doses are suppressive,^72^, ^73^ and suppression can be overcome by increasing the antigen dose.^17^ Moreover, low doses of IgG2a can enhance while high doses suppress anti‐SRBC responses.^106^

The outcome of feedback regulation can vary depending on which type of immune response is studied. An interesting discrepancy is the influence of antibody feedback on induction of CD4^+^ T cells. Complete suppression of antibody responses by IgG has little effect on T helper cells and enhancement of antibody responses by IgM and IgG3 is not accompanied by an enhancement of T helper cells. By contrast, IgE‐ and IgG2a‐mediated enhancement of antibody responses is paralleled by activation and proliferation of CD4^+^ T cells.

## 5 CONCLUDING REMARKS

My current understanding of antibody feedback regulation is that it is primarily a matter of antigen handling rather than central regulation of B‐cell activation. Most available data indicate that an important mechanism behind suppression is epitope masking. This requires that the antibodies bind to the antigen with high enough affinity to out‐compete specific B cells. Enhancement on the other hand is most likely caused by redistribution of antigen in a favorable way, either for presentation to B cells via FDC (IgM and IgG3) or to CD4^+^ T cells via dendritic cells (IgE, IgG1, IgG2a, and IgG2b). IgE–antigen complexes are captured by circulating CD23^+^ B cells which transport them to B‐cell follicles. Subsequently, antigen is taken up by CD8α^+^ conventional dendritic cells and presented to CD4^+^ T cells. IgM– and IgG3–immune complexes must be opsonized with complement in order to be deposited on FDC in B–cell follicles. IgG1, IgG2a, and IgG2b in complex with protein antigens require activating FcγRs in order to enhance. Complexes are captured by dendritic cells and presented to CD4^+^ T cells. In spite of these direct effects on antigen handling, it cannot be excluded that negative or positive B‐cell signaling via, for example, FcγRIIB or CR1/CR2 may fine‐tune in vivo immune responses, but that such effects are hidden in current experimental settings.

## CONFLICT OF INTEREST STATEMENT

The author have no conflicts of interest to disclose.

## Data Availability

The data that support the findings of this study are available from the corresponding author upon reasonable request.
